# Turing pattern formation on the sphere is robust to the removal of a hole

**DOI:** 10.1007/s00285-023-02034-z

**Published:** 2024-01-31

**Authors:** Johannes G. Borgqvist, Philip Gerlee, Carl Lundholm

**Affiliations:** 1 Wolfson Centre for Mathematical Biology, Mathematical Institute, University of Oxford, Andrew Wiles Building Radcliffe Observatory Quarter (550) Woodstock Road, Oxford, Oxfordshire OX2 6GG UK; 2 Mathematical Sciences, University of Gothenburg, Chalmers tvärgata 3, 412 96 Gothenburg, Västra Götaland Sweden; 3 Mathematical Sciences, Chalmers University of Technology, Chalmers tvärgata 3, 412 96 Gothenburg, Västra Götaland Sweden; 4 Department of Mathematics and Mathematical Statistics, Umeå University, MIT Building, 3rd Floor Linneaus Väg, 907 36 Umeå, Västerbotten Sweden

**Keywords:** Turing patterns, RD-models, Bud scars, FEM, 92B05, 35B36

## Abstract

The formation of buds on the cell membrane of budding yeast cells is thought to be driven by reactions and diffusion involving the protein Cdc42. These processes can be described by a coupled system of partial differential equations known as the Schnakenberg system. The Schnakenberg system is known to exhibit diffusion-driven pattern formation, thus providing a mechanism for bud formation. However, it is not known how the accumulation of bud scars on the cell membrane affect the ability of the Schnakenberg system to form patterns. We have approached this problem by modelling a bud scar on the cell membrane with a hole on the sphere. We have studied how the spectrum of the Laplace–Beltrami operator, which determines the resulting pattern, is affected by the size of the hole, and by numerically solving the Schnakenberg system on a sphere with a hole using the finite element method. Both theoretical predictions and numerical solutions show that pattern formation is robust to the introduction of a bud scar of considerable size, which lends credence to the hypothesis that bud formation is driven by diffusion-driven instability.

**Supplementary Information:**

The online version contains supplementary material available at 10.1007/s00285-023-02034-z.

## Introduction

A fascinating class of biological phenomena accounting for the emergence of complex patterns is that of *reaction diffusion* (RD) processes. Using two simple principles corresponding to chemical reactions between proteins and their movement due to diffusion, complex patterns in the concentration profile of these proteins emerge for certain types of reactions, and this pattern formation is often described by a class of *partial differential equations* (PDEs) which we will refer to as RD-models. The theoretical basis for this phenomenon, that was initially proposed by Turing (1952), is called *diffusion-driven instability* (Murray 2003), and based on this phenomenon RD-models have been applied in numerous situations including that of patterns in animal coatings and among fish (Watanabe and Kondo 2015), to name but a few.

**Fig. 1:**
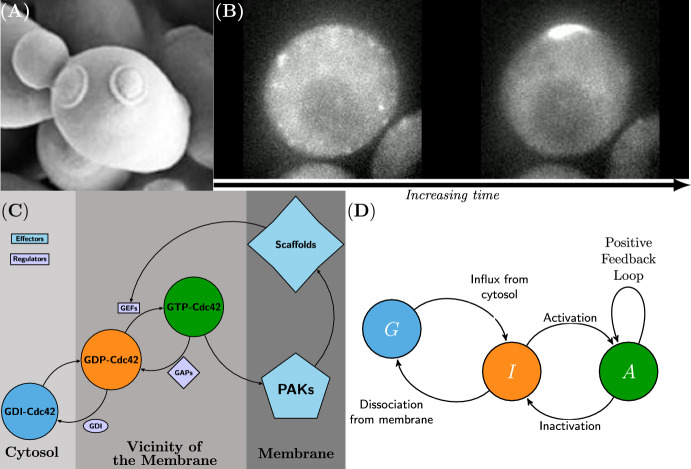
Cdc42 polarisation during the cell division of budding yeast. **A** Cell division known as budding in the baker’s yeast *Saccharomyces cerevisiae*: a smaller daughter cell grows out of the larger mother cell which eventually buds of resulting in two separate cells. Each time a division occurs a bud scar is left on the membrane of the mother cell. The mother cell in the figure has two clearly visible bud scars. **B** Cdc42-mediated cell polarisation in yeast. In the late G1-phase of the cell cycle prior to the budding event, activated Cdc42 accumulates at a particular spot on the cell membrane. This high concentration region is known as a pole and it is where the new daughter cell will eventually grow out. Here, Cdc42 has been tagged with a fluorescent dye. Initially there is no clear high concentration region of Cdc42 (left), whereas at a later time a pole has been formed (right). **C** A reaction mechanism for Cdc42 activation adapted from Fig. 1c in Borgqvist et al. (2021). The two membrane bound forms of Cdc42 are given by the activated form of Cdc42 (green), which is deactivated by the class of enzymes referred to as GAPs, and the inactivated form (orange), which is activated by the class of enzymes referred to as GEFs. There is a positive feedback loop [controlled by p21-activated kinases (PAKs) and polarity scaffold proteins (Chiou et al. 2017)] reinforcing the activation of Cdc42. There is also a cytosolic GDI bound state of Cdc42 which diffuses within the cell to the cell membrane. **D** An activator-inhibitor model of Cdc42-mediated polarisation based on the previously described reaction mechanism. This subfigure is adapted from Fig. 1a in Borgqvist et al. (2021) (color figure online)

Diffusion-driven instability has also been suggested to operate on the intra-cellular scale, in the context of cell division in the baker’s yeast *Saccharomyces cerevisiae*, also referred to as *budding yeast*. This type of yeast divides through a process called budding (Fig. 1A), where a smaller daughter cell grows out of the larger mother cell. After each division a so called *bud scar* is left on the cell membrane where the daughter cell budded of from the mother cell. The spatial location of the bud scar is determined by a protein called Cdc42 which is a so called GTPase (of the Rho family to be specific) which is an enzyme that has an active form when it is bound to a GTP molecule and an inactive form when it is bound to a GDP molecule. In the G1-phase of the cell cycle prior to the budding event, the activated form of Cdc42 diffuses on the cell membrane and eventually accumulates at a specific spot called a *pole* (Fig. 1B) being a high concentration region of Cdc42 on the cell membrane. It is at the pole that budding occurs and where the daughter cell ultimately grows out from the mother cell during the cell division. Moreover, the proteins that activate and inactivate Cdc42 are called GDP/GTP exchange factors (GEFs) and GTPase-activating proteins (GAPs), respectively (Chiou et al. 2017). It is the kinetics of GEFs and GAPs together with a positive feedback loop (Fig. 1C) in combination with the movement of Cdc42 due to diffusion that ultimately result in the formation of a pole. Besides, there are two particularly interesting aspects of Cdc42-mediated cell polarisation in the budding yeast *Saccharomyces cerevisiae* when it comes to the positioning of the poles and the bud scars on the cell membrane.

Firstly, bud scars tend to cluster on the cell membrane over successive divisions. The clustering of bud scars, meaning that new bud scars are formed close to already existing ones, is explained by so called landmarks proteins (Chiou et al. 2017) that are inherited from the mother cell. Their presence lead to the recruitment of extra GEFs around the bud scars, ultimately promoting the clustering of bud scars. Secondly, a new pole is never formed within an existing bud scar. More specifically, a pole is neither formed in the latest bud scar due to a specific GAP called Rga1 nor is it formed in any previous bud scars due to two landmarks proteins called Rax1 and Rax2 which block budding in previous bud scars (Chiou et al. 2017). Thus, a bud scar is a circular region on the cell membrane in which Cdc42 cannot polarise, and the positioning of bud scars is controlled by the local reinforcement of GEFs and GAPs which, in turn, is controlled by heritable landmark proteins.

The process of pole formation is thought to be described by an RD-process confined to the membrane of the cell. There are numerous RD-models of Cdc42-mediated cell polarisation (Borgqvist et al. 2021; Goryachev and Pokhilko 2008; Jilkine et al. 2007) and these are all of activator-inhibitor type (Fig. 1D). This means that Cdc42 is shuffled between its active and inactive forms through chemical reactions, and these reactions in combination with the diffusion of the two forms of Cdc42 results in the formation of a pole due to diffusion-driven instability. In one of the more complicated bulk surface models of Cdc42 activation based on the reaction schemes in Fig. 1C, D (Borgqvist et al. 2021), the key part of the involved equations enabling Turing patterns is the term modelling the positive feedback loop. Moreover, the simplest canonical activator-inhibitor RD-model which can form patterns is called the Schnakenberg model (Schnakenberg 1979), and this model is similar to the more complex bulk surface model of Cdc42 activation in Borgqvist et al. (2021) as it shares the term modelling the positive feedback loop. Due to its simplicity and capacity for forming Turing patterns, the Schnakenberg model is frequently used for both theoretical and numerical analysis of pattern formation caused by diffusion-driven instability.

One of the challenges when studying diffusion-driven instability for activator-inhibitor RD-models is the gap between the theoretical predictions and numerical simulations. In general, the theoretical formulae based on linear stability analysis for diffusion-driven instability are limited to relatively low-dimensional as well as geometrically simple domains, whereas simulations typically can account for a higher geometrical complexity. For example, it is not uncommon that the linear stability analysis is conducted in one spatial dimension, e.g., on a line, and then these theoretical results guide RD-simulations on a geometrical approximation of the cell in three spatial dimensions. This is problematic since there is a difference in geometry between the theoretical and numerical spatial domains, both in terms of dimensionality and curvature.

The simplest non-trivial spatial domain approximating the surface of a cell is the sphere, where theoretical predictions in combination with simulations have been conducted using the Schnakenberg model by Chaplain et al. (2001). In that context, it was possible to derive theoretical thresholds for a critical rate of diffusion as well as a critical reaction strength parameter which could be used to predict the properties of the resulting pattern. In fact, for the sphere and other curved manifolds such as prolate ellipsoids (see Krause et al. (2021) for a thorough review), the classical model of diffusion described by the standard Laplacian is instead captured by the Laplace–Beltrami operator expressed in appropriate coordinates on the manifold. A consequence of this is that the stability analysis conducted on such curved manifolds is more or less identical to the one conducted in the case of the standard Laplacian, although the spectrum and hence mode selection and final patterns differ (Krause et al. 2021).

However, for more irregular manifolds on which the spectrum cannot be explicitly calculated, linear stability analysis cannot be utilised. A possible solution to this problem is to consider the irregular manifold as a perturbation of a manifold with a known spectrum, and make use of results from spectral theory that allow for the calculation of the perturbed eigenvalues (Chavel and Feldman 1988).

From a mathematical point of view, introducing a bud scar on the surface of a spherical budding yeast cell can be modelled as such an irregular manifold. Since a bud scar is a region on the cell membrane where no activated Cdc42 can diffuse into, we can model it by a hole on the sphere together with *homogeneous Neumann boundary conditions*, i.e., no diffusive flux, on the boundary of the hole. The spectrum of the Laplace–Beltrami operator on the sphere with a hole cannot be explicitly calculated and therefore we make use of a perturbation expansion of the eigenvalues (Bandle et al. 2019). In addition, previously applied numerical methods [e.g., spectral methods of lines as used in Chaplain et al. (2001)] are unable to predict pattern formation in this case. With this in mind we set out to combine theoretical results from spectral theory with a finite element method (FEM) for solving the Schnakenberg system on the sphere with a hole, in order to describe the impact of bud scars on bud formation.

In this work, we have theoretically and numerically analysed the effect of a hole on the unit sphere $S^2$ on the pattern formation of the Schnakenberg model. The emergence of patterns is controlled by the eigenvalues of the Laplace–Beltrami operator on the sphere with a hole, and we have made use of results from spectral theory to calculate approximate values of the eigenvalues as a function of the radius of the hole. The theoretical results show that the patterns that appear on the sphere are retained although a hole of considerable size is introduced. This observation was verified by solving the Schnakenberg system numerically using the finite difference scheme 1-SBEM (Madzvamuse 2006) in time together with the finite element method in space. Our results show that diffusion-driven bud formation appears to be robust to the presence of bud scars.

## Results

### Validation of the numerical method

To study the pattern formation on a sphere we consider the Schnakenberg model. In its dimensionless form, this model is given by the following RD-system:

1 $$\begin{aligned} \begin{aligned} \left. \begin{array}{ll} \dfrac{\partial u}{\partial t} &{}= \Delta u + \gamma \left( a-u+u^2v\right) \\ \dfrac{\partial v}{\partial t} &{}= d \Delta v + \gamma \left( b-u^2v\right) \end{array}\right\} \quad&\text {in } S^2 \times (0, T], \\ u = u_0, v = v_0 \quad&\text {in } S^2 \times \{0\}. \end{aligned} \end{aligned}$$

Here, $S^2$ is the unit sphere and $T>0$ is a given final time. For ${\textbf{x}}\in S^2$ and $t \in [0,T]$, $u({\textbf{x}},t)$ is the concentration profile of the activator and $v({\textbf{x}},t)$ that of the inhibitor, $\Delta$ is the Laplace–Beltrami operator on $S^2$, $a,b,\gamma ,d>0$ are positive constants, and $u_0, v_0$ are given initial data. To numerically solve (1) and thus simulate pattern formation, we implemented a numerical method that uses the finite difference scheme 1-SBEM (Madzvamuse 2006) in time together with the finite element method in space. To validate the numerical method (see Sect. 4.4 in Methods) and its implementation, we replicated the results of Chaplain et al. [see figure 4.3 in Chaplain et al. (2001)] where the dynamics of the Schnakenberg model (1) on the sphere was solved numerically. Specifically, in these simulations, the following parameters were used:

2 $$\begin{aligned} a=0.20,\quad b=1.00,\quad \gamma =20.62,\quad d=18.00. \end{aligned}$$

**Fig. 2:**
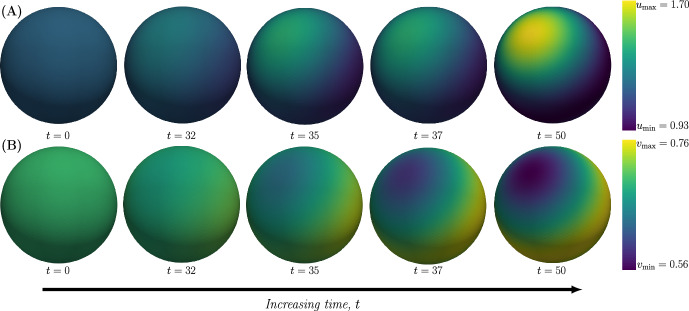
Validation of the implementation of the numerical method. The Schnakenberg model is simulated on the unit sphere $S^2$ with the following parameter values: $(a,b,d,\gamma )=(0.20,1.00,18.00,20.62)$. The time is increasing from left to right, and the concentration profiles at the time points $t=0$, $t=32$, $t=35$, $t=37$ and $t=50$ are shown in two cases. **A** The active component $u({\textbf{x}},t)$ with a minimum concentration of $u_{\min }=0.93$ and a maximum concentration of $u_{\max }=1.70$. **B** The inactive component $v({\textbf{x}},t)$ with a minimum concentration of $v_{\min }=0.56$ and a maximum concentration of $v_{\max }=0.76$. The time scale and the concentration ranges of the two species agree with Fig. 4.3 in Chaplain et al. (2001)

These values were chosen so that $n=2$ is the only mode in the unstable interval (see Sect. 4.2 in Methods for the definition of the unstable interval and how the values of *d* and $\gamma$ were chosen). For these parameter values, the steady-states are given by

3 $$\begin{aligned} u_0=a+b=1.20,\quad v_0=\frac{b}{(a+b)^2}=0.69 \end{aligned}$$

and starting from these initial conditions Chaplain et al. ran the simulations until a dimensionless time of $t=50$ was reached. Using the same exact parameter values, we validated our implementation by reproducing the results in Chaplain et al. (2001) [compare Fig. 2 with Fig. 4.3 in Chaplain et al. (2001)].

Given the validation of our numerical implementation, we now proceed to the problem of pattern formation on the sphere with a hole.

### Robustness of Turing patterns on the sphere with a hole: predictions from spectral theory

We model the addition of a bud scar on the cell membrane by considering the unit sphere $S^2$ with a hole. More precisely we consider a spherical cap $\Omega _{\varepsilon }$ centred at the North Pole $(0,0,1)\in {\mathbb {R}}^3$ of geodesic radius $\pi -\varepsilon$. Here $\varepsilon >0$ correspond to the geodesic radius of the hole, and we denote the boundary of the hole by $\partial \Omega _{\varepsilon }$.

It is known that as budding yeast cells go through multiple cell division the bud scars tend to aggregate on the cell membrane. Here we only model the addition of a single bud scar, but given their vicinity in real cells (Chiou et al. 2017) we model the accumulation of bud scars by considering a single hole of increasing radius $\varepsilon$.

Since the formation of Turing patterns is determined by the spectrum of the Laplace–Beltrami operator (see Sect. 4.1 in Methods for details), we are interested in the following eigenvalue problem

4 $$\begin{aligned} {\left\{ \begin{array}{ll} {}-\Delta Y_{n,\varepsilon }^m&{}=\lambda _n^m(\varepsilon ) Y_{n,\varepsilon }^m\quad \textrm{in}\quad \Omega _{\varepsilon }\subset S^2\subset {\mathbb {R}}^3\\ \partial _{{\textbf{n}}}Y_{n,\varepsilon }^m&{}=0\quad \textrm{on}\quad \partial \Omega _{\varepsilon } \end{array}\right. } \end{aligned}$$

where $Y_{n,\varepsilon }^m$ are the eigenfunctions of the Laplace–Beltrami operator on $\Omega _\varepsilon$, $\lambda _n^m(\varepsilon )$ are the corresponding eigenvalues, and $\partial _{{\textbf{n}}}$ is the derivative in the direction of the outer normal. Here we consider homogeneous Neumann boundary conditions, i.e., no diffusive flux, meant to describe a situation where no proteins can enter the bud scar.

**Fig. 3:**
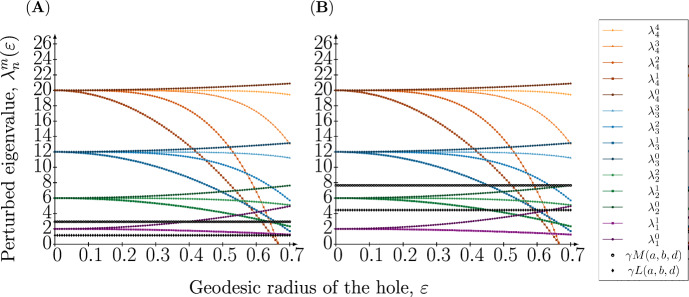
Perturbed eigenvalues as a function of the geodesic radius of the hole on the sphere. The perturbed eigenvalues $\lambda _n^m(\varepsilon )$ are plotted against the hole radius $\varepsilon$ when $n=1,2,3,4$ and $m=0,1,\ldots ,n$. Also, the upper boundary $\gamma M(a,b,d)$ and the lower boundary $\gamma L(a,b,d)$ in the Turing condition involving the eigenvalues giving rise to patterns are illustrated in the dashed lines. The parameters defining these boundaries are chosen to $(a,b)=(0.20,1.00)$ and the value of $\gamma$ is set to the critical value, i.e., $\gamma =\gamma _c(n)$ for a particular eigenmode *n*. The upper boundary $\gamma M(a,b,d)$ and the lower boundary $\gamma L(a,b,d)$ are illustrated in two cases: **A** $(n,\gamma ,d)=(1,6.87,20.00)$ and **B** $(n,\gamma ,d)=(2,20.62,18.00)$

**Fig. 4:**
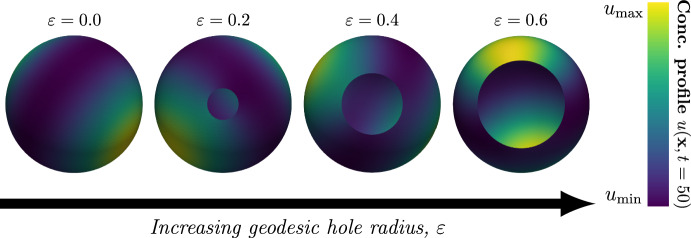
The pattern is robust with respect to the introduction of a hole in the domain. The concentration profile of the active component at time $t=50$, denoted by $u({\textbf{x}},t=50),\;{\textbf{x}}\in \Omega _{\varepsilon }$, is illustrated on four different meshes with a single hole located at the South Pole with geodesic radii $\varepsilon =0.00,0.20,0.40,0.60$. The parameters of the Schnakenberg model which were used to generate the above results were $(a,b,d,\gamma )=(0.20,1.00,18.00,20.62)$, and in all cases the initial conditions were set to a small perturbation around the steady-state concentrations of the two species in each node of the mesh. The maximum and minimum concentrations for the different geodesic hole radii $\varepsilon$ from left to right are given by: $(\varepsilon ,u_{\min },u_{\max })=(0.00,0.93,1.75)$, $(\varepsilon ,u_{\min },u_{\max })=(0.20,0.91,1.74)$, $(\varepsilon ,u_{\min },u_{\max })=(0.40,0.90,1.73)$ and $(\varepsilon ,u_{\min },u_{\max })=(0.60,0.93,1.6)$

Given this problem formulation, we are now interested in the eigenvalues $\lambda _n^m(\varepsilon )$ in (4) as a function of the radius of the hole $\varepsilon$. Asymptotic expansion of these eigenvalues in the limit of $\varepsilon \rightarrow 0$ have been derived by Bandle et al. (2019). These perturbed eigenvalues are given by Bandle et al. (2019):

5 $$\begin{aligned} \lambda _n^0(\varepsilon )=n(n+1)+(2n+1)\dfrac{4}{n(n+1)}\varepsilon ^2+o\left( \varepsilon ^2\right) ,\quad m = 0, \end{aligned}$$

and

6 $$\begin{aligned} \left. \begin{array}{ll} \lambda _n^m(\varepsilon )&{}=n(n+1)-(2n+1)c_n^m\varepsilon ^{2m}+o\left( \varepsilon ^{2m}\right) ,\\ c_n^m&{}=\dfrac{(m+n)!}{4^{m}\;m!\;(m-1)!\;(n-m)!} \end{array}\right\} ,\quad m=1,2,\ldots ,n. \end{aligned}$$

It is worth pointing out that the spectrum is continuous with respect to the hole radius and that $\lim _{\varepsilon \rightarrow 0} \lambda _n^m(\varepsilon ) = n(n+1)$, which equals the eigenvalues of the Laplace–Beltrami operator on the sphere.

In the limit of a small hole the formation of Turing patterns can thus be determined by considering a modified version of the unstable range of eigenvalues:

7 $$\begin{aligned} \gamma L(f_u,f_v,g_u,g_v,d)< \lambda _n^m(\varepsilon ) < \gamma M (f_u,f_v,g_u,g_v,d) \end{aligned}$$

where only eigenvalues/functions that fall in the above interval contribute to spatial patterning.

Figure 3 shows $\lambda _n^m(\varepsilon )$ as a function of $\varepsilon$ for $n=1$ and $n=2$, where lower and upper bounds in (7) are shown as dashed lines (see Sect. 4.2 in Methods for details behind parameter values). For $\varepsilon =0$ the eigenvalues for each *n* are degenerate, but as the radius of the hole increases they diverge, but remain within the pattern formation range for small values of $\varepsilon$. Thus we conclude that for these parameter settings, where a single eigenvalue lies in the unstable interval, we expect pattern formation to be unaffected by the introduction of a small hole.

We now move on to investigate this theoretical prediction using a numerical implementation of the Schnakenberg model on the sphere with a hole.

### Numerical solutions verify the theoretical prediction

To investigate the effect of a hole on pattern formation, and to test the above derived theoretical predictions, we considered the Schnakenberg model on the spherical cap $\Omega _{\varepsilon }$ together with homogeneous Neumann boundary conditions:

8 $$\begin{aligned} \begin{aligned} \left. \begin{array}{ll} \dfrac{\partial u}{\partial t} &{}= \Delta u + \gamma \left( a-u+u^2v\right) \\ \dfrac{\partial v}{\partial t} &{}= d \Delta v + \gamma \left( b-u^2v\right) \end{array}\right\} \quad&\text {in } \Omega _{\varepsilon } \times (0, T], \\ \partial _{{\textbf{n}}} u = 0, \partial _{{\textbf{n}}} v = 0 \quad&\text {on } \partial \Omega _{\varepsilon } \times (0, T], \\ u = u_0, v = v_0 \quad&\text {in } \Omega _{\varepsilon } \times \{0\}. \end{aligned} \end{aligned}$$

We refer to the previous two subsections for details. We solved (8) numerically on spheres with an increasing hole radius for four parameter sets, corresponding to patterns with a single excited mode ($n=1,2,3$ and 4). The rate parameters *a* and *b* were fixed to the values $a=0.20$ and $b=1.00$ in all simulations according to (2). We set the values of the reaction strength $\gamma$ and the relative diffusion *d* based on critical values such that a single mode was excited (see Sect. 4.2 in Methods for details).

An example of the effect of increasing the hole radius is shown in Fig. 4, where the mode $n=2$ is excited and $\varepsilon =0$ corresponds to a complete sphere without a hole. From this it appears as if the addition of a hole has little effect on the resulting pattern.

In order to investigate this further, we varied the hole size determined by the geodesic radius $\varepsilon$ in the range $\varepsilon \in [0,0.7]$, and to account for the stochasticity of the solutions, which are introduced via the initial conditions, each simulation was repeated 20 times. We set out to characterise the resulting patterns both by projecting the solutions onto the spherical harmonics and by quantifying the number of poles, the maximum concentration of *u*, the relative area of the poles and the minimum distance from the hole to a pole (see Sects. 4.5, 4.6, 4.7 in Methods for details).

**Fig. 5:**
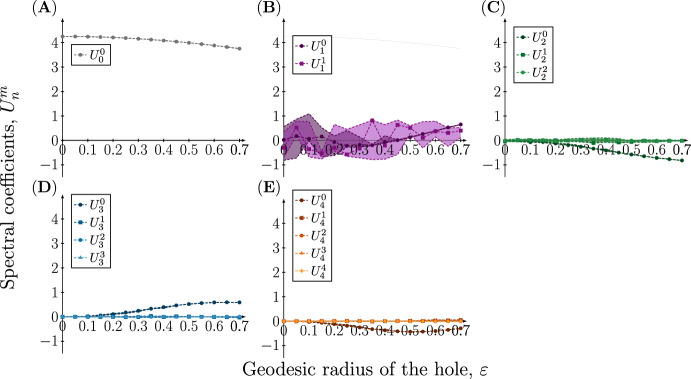
Spectral coefficients of the concentration profile of the active component at time $t=50$ on meshes with a single hole with increasing radius when $(n,d)=(1,20)$. The coefficients of the eigenfunctions $Y_n^m$ in the spectral approximation of the concentration profile $u({\textbf{x}},t=50),\;{\textbf{x}}\in \Omega _{\varepsilon }$ resulting from the rate parameters $(a,b,d,\gamma )=(0.20,1.00,20.00,6.87)$ are plotted as a function of the geodesic radius of the hole $\varepsilon$. Due to the stochasticity in the initial conditions, each simulation has been repeated 20 times, and to account for the variation in the coefficients the 95%, 50% and 5% percentiles are plotted

The projection of the numerical solution onto the spherical harmonics provides a way of investigating which modes are excited in the resulting pattern. Figure 5 shows the decomposition in the case where $n=1$ is excited. Here we observe large variability in the coefficients $U_1^0$ and $U_1^1$, whereas the other coefficients show less variability and take values close to zero. For $U_1^0$ there is a statistically significant dependence on the hole radius, but this is not the case for $U_1^1$ (see Sect. 4.8 in Methods). The constant mode $U_0^0$ takes a considerably larger value, but this value corresponds to the steady-state value from which the pattern emerges, and can be explicitly calculated in the following way: the zeroth mode is stable with respect to spatial perturbations and we therefore expect it to remain approximately constant as the dynamics evolve. Since it is constant we can express it in terms of the initial concentration $u(t=0,x)=u_0 = U_0^0 Y_0^0 = U_0^0 \frac{1}{2\sqrt{\pi }}$, and since $u_0=1.2$ we obtain $U_0^0 = 1.2 \cdot 2\sqrt{\pi } \approx 4.25$, which is close to the value observed for small $\varepsilon$ in Fig. 5A.

**Fig. 6:**
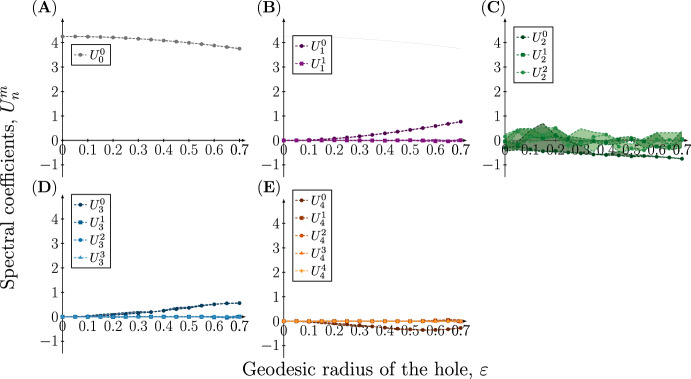
Spectral coefficients of the concentration profile of the active component at time $t=50$ on meshes with a single hole with increasing radius when $(n,d)=(2,18)$. The coefficients of the eigenfunctions $Y_n^m$ in the spectral approximation of the concentration profile $u({\textbf{x}},t=50),\;{\textbf{x}}\in \Omega _{\varepsilon }$ resulting from the rate parameters $(a,b,d,\gamma )=(0.20,1.00,18.00,20.62)$ are plotted as a function of the geodesic radius of the hole $\varepsilon$. Due to the stochasticity in the initial conditions, each simulation has been repeated 20 times, and to account for the variation in the coefficients the 95%, 50% and 5% percentiles are plotted

The case where $n=2$ is excited for the sphere is shown in Fig. 6. Here we observe a similar pattern with variation in the coefficients corresponding to the excited mode and all other coefficients remaining small, with the exception of $U_0^0$. For $U_2^0$ there is a statistically significant dependence on the hole radius, whereas this is not the case for $U_2^1$ and $U_2^2$. The pattern is repeated for $n=3$ and 4 (see Supplementary information), and we thus conclude that variation occurs only in the mode that was excited for the sphere and that introducing a hole has a minor effect on other modes.

**Fig. 7:**
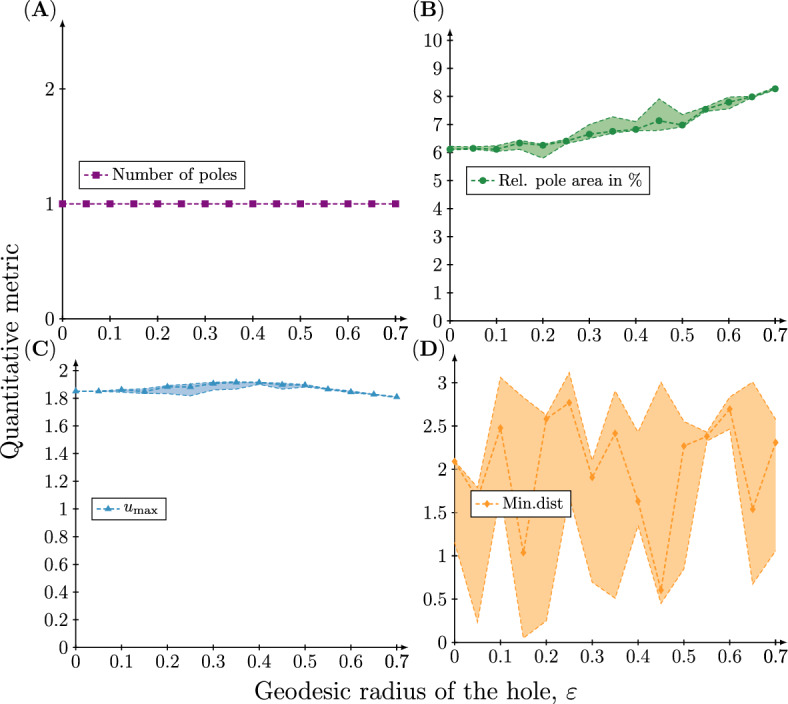
Quantitive metrics of the concentration profile of the active component at time $t=50$ on meshes with a single hole with increasing radius when $(n,d)=(1,20)$. Four different quantitative metrics of the concentration profile $u({\textbf{x}},t=50),\;{\textbf{x}}\in \Omega _{\varepsilon }$ resulting from the rate parameters $(a,b,d,\gamma )=(0.20,1.00,20.00,6.87)$ are plotted as a function of the geodesic radius of the hole $\varepsilon$. **A** The number of poles corresponding to high concentration regions. **B** The total pole area relative to the total surface area. **C** The maximum concentration $u_{\max }$. **D** The minimal *great circle distance* between a pole and the hole. Each simulation has been repeated 20 times and therefore the 95%, 50% and 5% percentiles of the quantitative metrics are plotted

**Fig. 8:**
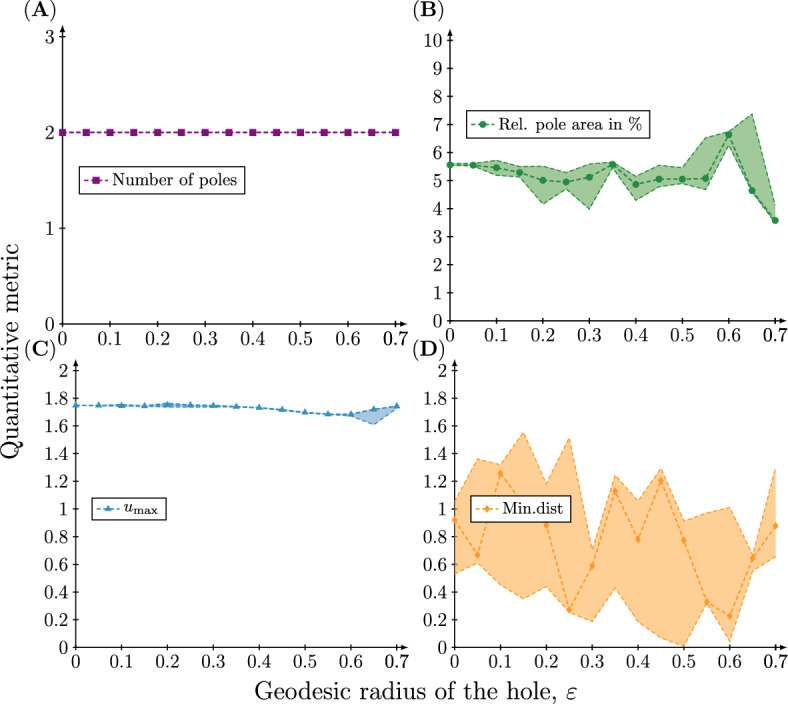
Quantitive metrics of the concentration profile of the active component at time $t=50$ on meshes with a single hole with increasing radius when $(n,d)=(2,18)$. Four different quantitative metrics of the concentration profile $u({\textbf{x}},t=50),\;{\textbf{x}}\in \Omega _{\varepsilon }$ resulting from the rate parameters $(a,b,d,\gamma )=(0.20,1.00,18.00,20.62)$ are plotted as a function of the geodesic radius of the hole $\varepsilon$. **A** The number of poles corresponding to high concentration regions. **B** The total pole area relative to the total surface area. **C** The maximum concentration $u_{\max }$. **D** The minimal *great circle distance* between a pole and the hole. Each simulation has been repeated 20 times and therefore the 95%, 50% and 5% percentiles of the quantitative metrics are plotted

We now turn to our quantitative metrics of pattern formation. Figure 7A shows that for $n=1$ the number of poles that are present at $t=50$ in the numerical solution is constant and equal to one for the entire range of hole sizes. Also the relative pole area (Fig. 7B) and the maximum concentration of *u* (Fig. 7C) are preserved. In contrast, the distance from the centre of the hole to the closest pole varies considerably, but there is no statistically significant dependence between the hole radius and the minimal distance. Precisely the same pattern is observed for $n=2$ (see Fig. 8) (similar results for $n=3$ and 4 can be found in the Supplementary information). Again, there is no statistically significant dependence between the hole radius and the minimal distance.

These results show that the introduction of a hole does not affect the resulting pattern. The variation in distance between the hole and closest pole appears simply because the orientation of the pattern depends on the random initial conditions and thus varies between simulations. This also explains the variation observed in the spectral coefficients of the excited mode. The orientation of the pattern causes the coefficients of the excited modes that constitute the pattern to vary, when in fact the pattern (modulo rotation) is preserved.

## Discussion

In this work, we have investigated the effect of introducing a hole of varying size on the unit sphere on the Turing patterns exhibited by the Schnakenberg model. Using a FEM-based implementation together with a spectral analysis in terms of the excited eigenfunctions of the Laplace–Beltrami operator contributing to the solution, we concluded that the quantitive properties of the final patterns are largely conserved when a hole is introduced.

This observation can be in part explained by considering an asymptotic expansion of the eigenvalues of the Laplace–Beltrami operator on the sphere with a hole. In the limit of small hole sizes, the perturbed eigenvalues can be written as a power series in the hole radius $\varepsilon$. This implies that the eigenvalues are continuous with respect to the hole radius and that the eigenvalues that are in the unstable range for the sphere (without a hole), remain so even in the presence of small hole (see Fig. 3). The opposite also holds true, for small $\varepsilon$ no other eigenvalues enter the unstable region, and thus the pattern is conserved.

The conclusion hinges on the continuity of the spectrum of the Laplace–Beltrami operator with respect to the removal of a small circular region of the sphere. Despite the fact that the topology of the domain is altered, the spectrum is continuous and $\lim _{\varepsilon \rightarrow 0} \lambda _n^m(\varepsilon ) = \lambda _n^m$. This property holds for a much wider class of perturbations of the domain, as has been proven by Chavel and Feldman (1988), and in particular the addition of finitely many holes on the sphere. However, explicit formulae for the perturbed eigenvalues have only, in the more general setting, been derived for Dirichlet boundary conditions. In the reaction diffusion setting, considered here, it makes less sense to consider such boundary conditions, yet we conjecture that pattern formation in the Schnakenberg model (with Neumann boundary conditions) will remain intact even in the case of finitely many holes of small radius.

There are are a number limitations to our approach and the conclusions drawn from it. In order to analyse the resulting pattern we project the numerical solutions on the sphere with a hole onto the spherical harmonics, although they do not form an orthonormal ${\mathcal {L}}_2$ basis for the domain. However, we expect this to be a reasonable approximation, in particular for small hole sizes. This implies that the spectral decomposition shown in Figs. 5 and 6 should be interpreted with caution, especially for hole sizes in the upper range. This might also be the reason why we observe a statistically significant dependence of the hole radius on some spectral coefficients, e.g., in Fig. 5B the increase in $U_1^0$ occurs only for larger hole radii.

The asymptotic expansions obtained from Bandle et al. (2019) are only valid in the limit of small hole sizes. This means that our conclusions regarding which eigenvalues that fall within the unstable region in (7) are only strictly valid in the limit of small hole sizes. Further, it implies that the plots showing the relation between perturbed eigenvalues and the unstable region (Fig. 3) are not accurate for large hole sizes.

Motivated by the fact that bud scars of ageing yeast cells tend to cluster (Chiou et al. 2017), we have considered a hole of increasing radius meant to represent several smaller bud scars. This is a crude approximation, but the theoretical results mentioned above suggest that the effect of several small holes on the eigenvalues of the Laplace–Beltrami operator should have a similar impact as a single large hole.

Our results suggest that bud formation in budding yeast is robust to changes that alter the topology of the membrane on which the reactions that drive the accumulation of Cdc42 occur. Bud formation corresponds to an instability of the eigenvalue $n=1$ whose eigenfunction contains a single peak, but we have shown that patterns corresponding to $n=1,2,3$ and 4 are stable with respect to the addition of a hole as well.

From a biological stand point this is far from surprising since budding yeast cells are known to form many buds (and thus contain many bud scars) before they reach senescence and stop dividing. From a mathematical modelling point of view our results lend credence to the idea that activator-inhibitor dynamics (as modelled by the Schnakenberg model) are a good model of the process of bud formation. If the dynamics were highly sensitive to the addition of a hole they would indeed present a poor model of the phenomenon.

In relation to this it should be noted that budding yeast cells of advanced age are dysfunctional and tend to form multiple buds (Ishihara et al. 2007; Chiou et al. 2018, 2021). This can in part be explained by the fact that cell size increases with age, and in the non-dimensional Schnakenberg model this corresponds to an increase in the reaction strength $\gamma$. Mathematically, this leads to a widening of the unstable region in the spectrum, which might cause additional eigenmodes to become excited leading to patterns with multiple poles. Additionally, it has been hypothesised that oxidative stress alters the enzymatic activity of the proteins that move Cdc42 between its active and inactive form. In terms of the Schnakenberg model this would correspond to changing the reaction parameters *a* and *b*, which could disrupt the pattern forming properties of the system. However, as we have shown, the addition of a hole perturbs the eigenvalues, which might contribute to additional eigenmodes being excited. Thus, it might be both the increasing cell size, altered enzymatic activity and a large number of bud scars that contribute to dysfunctional budding.

A feature not captured by our model is that new buds tend to form close to existing bud scars (Chiou et al. 2018). This could be the result of space dependent reaction rates (Page et al. 2003), which could be introduced into our model. Further, it would be of interest to investigate if other RD-models such as the Thomas model (Murray 2001) and the Gray–Scott model (Gray and Scott 1984) are equally robust to the addition of holes on the sphere.

In conclusion, we have shown that RD-dynamics for pole formation in budding yeast can be modelled with a finite element approach, which makes it possible to account for the presence of an existing bud scar of varying size. This method is highly flexible and can be adapted to solve RD-models on any domain that can be represented by a mesh. Numerical solutions show that the introduction of a bud scar does not affect the resulting pattern, and this can in part be explained by appealing to results from spectral theory, that describe how the eigenvalues of the Laplace–Beltrami operator are perturbed in the presence of a hole. The stability of pole formation with respect to the addition of bud scars add theoretical evidence for pole formation being a Turing pattern of activator-inhibitor type, but more research in this direction is needed to explain dysfunctional pole formation in ageing cells and also the clustering of bud scars on the cell membrane.

## Methods

All the scripts for generating the results presented in this work can be accessed through the public GitHub repository associated with this work (Borgqvist et al. 2023). The entire project is written in Python and it only uses open-source dependencies. To visualise these results and to generate the figures, the software ParaView (Ahrens et al. 2005; Ayachit 2015) has been used.

### Turing patterns on the sphere

It has been hypothesised that the formation of buds in budding yeast, which initiates the formation of a daughter cell and subsequent cell division, is driven by reaction and diffusion of molecules that are confined to the cell membrane. The spatial distribution of chemical species on the membrane can be described by a coupled set of partial differential equations known as reaction-diffusion equations. The formation of spatial patterns in such systems is known as a Turing instability and can appear under specific constraints on the diffusion coefficients and reaction rates of the involved chemical species.

A reaction–diffusion system of two interacting species *u* and *v* on the sphere $S^2$ can be written in non-dimensonal form as

9 $$\begin{aligned} \begin{aligned} \dfrac{\partial u}{\partial t}&= \Delta u + \gamma f(u,v) \\ \dfrac{\partial v}{\partial t}&= d \Delta v + \gamma g(u,v), \end{aligned} \end{aligned}$$

where *d* is the ratio of the (dimensional) diffusion coefficients, *f*, *g* the reaction rates, $\gamma$ is a constant related to the radius and $\Delta$ is the Laplace–Beltrami operator on $S^2$.

A homogeneous steady state $(u_0,v_0)$ exhibits diffusion-driven Turing instability (Murray 2003; Chaplain et al. 2001) if it satisfies $f(u_0,v_0)=g(u_0,v_0)=0$ and the following inequalities are satisfied

10 $$\begin{aligned} f_u + g_v < 0,{} & {} \det (A) > 0, \end{aligned}$$

11 $$\begin{aligned} df_u + g_v>0,{} & {} (df_u+g_v)^2-4d\det (A) >0. \end{aligned}$$

Here $f_u, g_v$ are partial derivatives evaluated at $(u_0,v_0)$, and *A* is the Jacobian, also evaluated at the steady state. The type of pattern that appears depends on the range of unstable modes in the spectrum of the Laplace–Beltrami operator. The Laplace–Beltrami eigenvalue problem on the sphere is:

12 $$\begin{aligned} -\Delta Y_n^m=\lambda _n Y_n^m,\quad \lambda _n=n(n+1)\quad \textrm{in}\quad \Omega =S^2\subset {\mathbb {R}}^3. \end{aligned}$$

Here, the eigenfunctions are the spherical harmonics $Y_n^m$, where $\vert m \vert \le n$. A mode *n* is unstable if it falls in the range (Murray 2003; Chaplain et al. 2001)

13 $$\begin{aligned} \gamma L(f_u,f_v,g_u,g_v,d)< \lambda _n=n(n+1) < \gamma M (f_u,f_v,g_u,g_v,d) \end{aligned}$$

where

14 $$\begin{aligned} L = \frac{df_u+g_v - \sqrt{(df_u+g_v)^2-4d\det (A)}}{2d} \end{aligned}$$

and

15 $$\begin{aligned} M=\frac{df_u+g_v + \sqrt{(df_u+g_v)^2-4d\det (A)}}{2d}. \end{aligned}$$

If there exists at least one such *n* then the homogeneous steady state is unstable with respect to spatial perturbations and we can expect a heterogeneous pattern composed of the eigenfunctions $Y_n^m$, $\vert m \vert \le n$, that satisfy (13).

In summary, provided that the parameters in the reaction terms *f*, *g* of the RD-model (9) are chosen so that the Turing conditions (10) and (11) are satisfied, it is the spectrum of the Laplace–Beltrami operator that determines pattern formation. More specifically the eigenmodes *n* that satisfy the bounds in (13).

### Finding critical values for $\gamma$ and *d*

In order to obtain diffusion-driven instability the diffusion coefficient *d* needs to be above a certain critical value, which is given by Chaplain et al. (2001):

16 $$\begin{aligned} d_c=\dfrac{(f_u g_v-2f_v g_u)+\sqrt{(f_u g_v-2f_v g_u)^2-f_u^2 g_v^2}}{f_u^2} \end{aligned}$$

where $f_u,f_v,g_u,g_v$ correspond to the partial derivatives in the Jacobian evaluated at the steady state. Only the eigenvalues that lie within the unstable interval contribute to pattern formation, and in order to isolate a single eigenvalue (and thus a single mode) one sets $\gamma$ to a critical value which is a function of the eigenvalue *n* (Chaplain et al. 2001):

17 $$\begin{aligned} \gamma _c(n)=\dfrac{2d_c n^2(n+1)^2}{d_c f_u+g_v}. \end{aligned}$$

For the parameters $a=0.20$ and $b=1.00$, the critical diffusion is given by $d_c\approx 17.01$. If we pick $\gamma =\gamma _c(n)$ for a specific eigenmode *n* and *d* sufficiently close to $d_{c}$, then the resulting concentration profile will be a linear combination of the eigenfunctions corresponding to the particular eigenmode *n* (in addition to the zeroth eigenmode) and no other eigenmodes. To this end, we have picked *d* sufficiently close to $d_c$ as well as the value $\gamma =\gamma _c(n)$ in the four cases corresponding to $n=1,2,3,4$, respectively, in our experiments.

### The mesh for geometrically approximating the sphere with a hole

The meshes were generated using Gmsh (Geuzaine and Remacle 2009). We generated our holes in the mesh by intersecting a cylinder with the sphere, and then we removed the intersection in order to obtain a mesh of the spherical cap.

Given these meshes, we implemented our FEM-based numerical scheme for solving the Schnakenberg model on a spherical mesh with a single hole located at the South Pole, i.e., centered at $(x,y,z)=(0,0,-1)$. A visualisation of the meshes can be found in the Supplementary information (Fig. S1).

### The numerical scheme for simulating Schnakenberg’s RD-model using a FEM-FD approach

We implemented the 1-SBEM numerical scheme (Madzvamuse 2006) for solving the Schnakenberg model in FEniCS (Alnæs et al. 2015; Logg et al. 2012). For all details behind this algorithm, we refer to Madzvamuse (2006), but here follows a short summary of the involved steps.

Given the Schnakenberg RD-model, the first step is to multiply the system of PDEs with two test-functions $\phi _1,\phi _2\in {\mathcal {H}}(\Omega _{\varepsilon }){:}{=}\big \{f({\textbf{x}}):\Vert f\Vert _{{\mathcal {L}}_2(\Omega _{\varepsilon })}+\Vert \nabla f\Vert _{{\mathcal {L}}_2(\Omega _\varepsilon )}<\infty ,\quad \forall {\textbf{x}}\in \Omega _{\varepsilon }\big \}$ where the ${\mathcal {L}}_2$-norm is induced by the inner product

$$\begin{aligned} \langle f,g\rangle _{{\mathcal {L}}_2(\Omega _{\varepsilon })}=\int _{\Omega _{\varepsilon }}f({\textbf{x}})g({\textbf{x}})\textrm{d}{\textbf{x}} \end{aligned}$$

and then integrate over the domain $\Omega _{\varepsilon }$. Given this operation, the RD-system is written as follows:

$$\begin{aligned} \begin{aligned} \left\langle \dfrac{\partial u}{\partial t},\phi _1\right\rangle _{{\mathcal {L}}_2(\Omega _{\varepsilon })}&=\langle \Delta u,\phi _1\rangle _{{\mathcal {L}}_2(\Omega _{\varepsilon })}+\gamma \langle f(u,v),\phi _1\rangle _{{\mathcal {L}}_2(\Omega _{\varepsilon })}\\ \left\langle \dfrac{\partial v}{\partial t},\phi _2\right\rangle _{{\mathcal {L}}_2(\Omega _{\varepsilon })}&=d\langle \Delta v,\phi _2\rangle _{{\mathcal {L}}_2(\Omega _{\varepsilon })}+\gamma \langle g(u,v),\phi _2\rangle _{{\mathcal {L}}_2(\Omega _{\varepsilon })}\\ \end{aligned} \end{aligned}$$

where the reaction terms *f*, *g* are given by (8). Using Green’s formula on the diffusive terms in combination with the fact that we have homogeneous Neumann boundary conditions on the boundary of the hole $\partial \Omega _{\varepsilon }$, we can move all terms to the left hand sides and then add the two equations. This results in one single equation which is our *variational formulation*:

18 $$\begin{aligned}{} & {} \text {Find}\,\, u(\cdot ,t),v(\cdot ,t)\in {\mathcal {H}}(\Omega _\varepsilon ) \,\, \text {for a fixed}\,\, t\in {\mathbb {R}}_+\,\, \text {such that}\nonumber \\{} & {} \quad \left\langle \dfrac{\partial u}{\partial t},\phi _1\right\rangle _{{\mathcal {L}}_2(\Omega _{\varepsilon })}+\left\langle \dfrac{\partial v}{\partial t},\phi _2\right\rangle _{{\mathcal {L}}_2(\Omega _{\varepsilon })}+\langle \nabla u,\nabla \phi _1\rangle _{{\mathcal {L}}_2(\Omega _{\varepsilon })}+d\langle \nabla v,\nabla \phi _2\rangle _{{\mathcal {L}}_2(\Omega _{\varepsilon })}\nonumber \\{} & {} \qquad -\gamma \langle f(u,v),\phi _1\rangle _{{\mathcal {L}}_2(\Omega _{\varepsilon })}-\gamma \langle g(u,v),\phi _2\rangle _{{\mathcal {L}}_2(\Omega _{\varepsilon })}=0. \end{aligned}$$

To find the numerical approximation, this variational formulation is restricted to a discrete subspace of continuous piecewise linear functions. This method is referred to as *continuous Galerkin of order 1* denoted by cG(1).

To approximate the time derivatives in (18), we use a finite difference scheme. Given that we want to solve the original RD system for times in the time interval (0, *T*] for some final time $T>0$, we define a partition ${\mathcal {T}}_k$ of the interval [0, *T*] into *N* pieces for some integer $N>0$ as follows:

$$\begin{aligned} {\mathcal {T}}_k=\left\{ t_i=ik,\quad k=\dfrac{T}{N}\quad \text {for}\quad i=0,1,2,\ldots ,N\right\} . \end{aligned}$$

Given this partition, the time derivatives in (18) are approximated by

$$\begin{aligned} \dfrac{\partial u}{\partial t}\bigg |_{t=t_i}\approx \dfrac{u(t_i)-u(t_{i-1})}{k},\quad \dfrac{\partial v}{\partial t}\bigg |_{t=t_i}\approx \dfrac{v(t_i)-v(t_{i-1})}{k} \end{aligned}$$

and then the defining feature of the 1-SBEM numerical scheme in Madzvamuse (2006) is how the reaction terms *f*, *g* in (18) are approximated. The approximations of the reaction terms are given by

19 $$\begin{aligned} \begin{aligned} f\left( u(t_{i-1}),v(t_{i-1}),u(t_{i}),v(t_{i})\right)&=a-u(t_i)+u(t_i)u(t_{i-1})v(t_{i-1}),\\ g\left( u(t_{i-1}),v(t_{i-1}),u(t_{i}),v(t_{i})\right)&=b-u(t_{i-1})^2v(t_{i}).\\ \end{aligned} \end{aligned}$$

This method is particularly clever as it separates the two unknowns which the FEM solves for in each time step, namely the concentration profiles of the states at the current time step given by $u(t_i)$ and $v(t_i)$. This means that in the resulting FEM-formulation, there will only be terms containing inner products between the approximation of the activator *u* and its corresponding test function $\phi _1$ as well as inner products between the approximation of the inhibitor *v* and its corresponding test function $\phi _2$. Therefore, there is no mixing between the two states and essentially this means that two separate matrix equations can be solved in each time step for both of the states simultaneously which increases the efficiency of the algorithm.

Here, it is important to emphasise that higher values of $\gamma$, require smaller step sizes *k* in the 1-SBEM-time-stepping procedure. By trial and error, we determined an appropriate step size giving rise to stable solutions for a given eigenmode *n* (Table 1) in the case when $\gamma$ was set to its critical value according to $\gamma =\gamma _c(n)$ (17).

**Table 1:** The step sizes *k* used in the 1-SBEM-time-stepping procedure as a function of the eigenmode *n*

Eigenmode, *n*	$\gamma =\gamma _c(n)$	Step size, *k*
1	6.87	0.01
2	20.62	0.01
3	41.24	0.005
4	68.73	0.005

### Numerical solutions

For each numerical solution we used initial conditions that were generated by randomly perturbing the steady states concentrations in (3) at every nodal point of the mesh by a value from a uniform distribution on $$[-10^{-4}, 10^{-4})$$. Then, we ran all simulations to a final time of $$T=50$$ with single hole located at the South Pole, i.e., at $$(x,y,z)=(0,0,-1)$$, with 15 different geodesic hole radii in the range $$r=0,\ldots ,0.70$$ where the radius $$r=0$$ corresponds to the sphere with no hole. To account for the stochasticity in the initial conditions, we repeated each simulation 20 times, and thus in total we ran

$$\begin{aligned} \underset{\text {Parameter sets}}{\underbrace{4}}\times \underset{\text {Hole sizes}}{\underbrace{15}}\times \underset{\text {Repititions}}{\underbrace{20}}~\textrm{simulations}=1200~\text {simulations}. \end{aligned}$$

Here, we want to emphasise that we ran a single simulation on a much longer time scale than the final time $T=50$ implemented throughout this work. Specifically, using the parameter values $(a,b,d,\gamma )=(0.20,1.00,20.00,6.87)$ we ran a single simulation until a final time of $T=500$ (see section S4.5 in Supplementary information) was reached. Despite this time scale being 10 times longer than the standard one implemented throughout this work, the concentration profile was identical on both time scales. Based on this, we concluded that the time scale $T=50$ is long enough to capture the long term dynamics of the system.

Using all the data that was generated from these simulations, we analysed two types of properties as functions of the increasing hole radius. Firstly, we approximated the final concentration profile of the active component *u* at time $t=50$ in terms of the first couple of eigenfunctions $Y_n^m$ of the Laplace–Beltrami operator (12) in order to deduce what eigenfunctions contributed to the solution. Secondly, we analysed four quantitative metrics of the concentration profile of the active component *u* at time $t=50$. More specifically, we calculated the number of poles where a pole corresponds to a high-concentration region, the maximum concentration $u_{\max }$, the area of the pole relative to the total area of the sphere, and the great-circle distance between the midpoint of the introduced hole and the closest pole.

### Spectral decomposition of the FEM solution by means of projection

We performed the spectral decomposition of the FEM solution by means of projection onto the spherical harmonics, which form an orthonormal basis for ${\mathcal {L}}_2(S^2)$. More precisely, let $u({\textbf{x}},t=50)$ be the FEM solution at time $t=50$ given by

20 $$\begin{aligned} u({\textbf{x}},t=50) =\sum _{i=1}^{N}c_i\phi _i({\textbf{x}}),\quad {\textbf{x}}\in \Omega _{\varepsilon } \end{aligned}$$

where *N* corresponds to the number of nodes in the mesh, $i=1,\ldots ,N$ is an index, $c_i$ are coefficients and $\phi _i$ are the piecewise linear basis functions. The idea is to express the FEM-solution above in terms of the spherical harmonics $Y_n^m$ according to

21 $$\begin{aligned} u({\textbf{x}},t=50) = \sum _{n=0}^{\infty }\sum _{m=-n}^{n}U_n^mY_n^m({\textbf{x}}),\quad {\textbf{x}}\in \Omega _{\varepsilon } \end{aligned}$$

where $U_n^m$ are the unknown coefficients which we wish to compute. We compute the coefficients $U_n^m$ by the following formula

22 $$\begin{aligned} U_n^m=\int _{\Omega _{\varepsilon }}u({\textbf{x}},t=50)Y_n^m({\textbf{x}})\;\textrm{d}{\textbf{x}} = {\left\{ \begin{array}{ll} \int _{\Omega _{\varepsilon }}u({\textbf{x}},t=50)Y_{n,0}({\textbf{x}})\;\textrm{d}{\textbf{x}},\quad &{}m=0\\ \dfrac{1}{\sqrt{2}}\int _{\Omega _{\varepsilon }}u({\textbf{x}},t=50)Y_{n,m}({\textbf{x}})\;\textrm{d}{\textbf{x}},\quad &{}m>0\\ \end{array}\right. }.\nonumber \\ \end{aligned}$$

In accordance with Chaplain et al. (2001), we have used the real part of the complex spherical harmonics and taken $Y_n^{-m} = Y_n^m$. The $Y_{n,m}$’s in (22) are thus the real spherical harmonics. A table of the $Y_{n,m}$’s for the eigenmodes $n=0,1,2,3,4,5$ can be found in the Supplementary information (Table S1). For brevity, we only calculate the coefficients $U_n^m$ of our FEM-solutions in terms of the first couple of eigenmodes *n*.

### Quantifying the number of poles using density based spatial clustering

To calculate the number of poles in an automated fashion, we used the Python based package for machine learning called *scikit-learn* (Pedregosa et al. 2011). More precisely, we used the function *DBSCAN*, which stands for *Density-Based Spatial Clustering*, in the following way.

Firstly, we extracted all spatial points ${\textbf{x}}\in \Omega _{\varepsilon }$ in the mesh that belonged to a pole in the concentration profile $u({\textbf{x}},t=50)$ which is a high–concentration region. In turn, a point ${\textbf{x}}\in \Omega _{\varepsilon }$ was classified as belonging to a pole if the concentration at that point in the mesh was greater or equal to 95% of the maximum concentration, i.e., if $u({\textbf{x}},t=50)\ge 0.95\;u_{\max }$. After all coordinates in the mesh that belonged to poles were extracted, the DBSCAN function was implemented in order to find out the number of clusters of spatial points which, in turn, corresponds to the number of poles.

Here, we want to emphasise that the threshold of 95% of the maximum concentration profile is arbitrary. The reason we picked this value was because the number of poles returned by DBSCAN agreed with that obtained by visual inspection.

In addition, the choice of the threshold value to $0.95\;u_{\max }$ determines the calculated area of the poles relative to the total surface area. This particular threshold value gives rise to a total pole area of approximately 8% of the total surface area. Moreover, a higher threshold value yields a lower total pole area while a lower threshold value yields a higher total pole area.

### Statistical tests

In order to investigate if there is a statistically significant dependence between the hole radius and the spectral coefficients we calculate the mean of the coefficients across the repetitions for each value of the hole radius. We then perform a linear regression with the hole radius as the independent variable and the average value of the coefficient as the dependent variable. Lastly, we carry out a two-sided *t* test at significance level 0.05 whose null hypothesis is that the slope is zero. The same procedure is carried out for the minimal distance between the pole and the hole.

## Supplementary information

For more results and details, we refer to the Supplementary information associated with this work. We would like to emphasise that the code is available at the public GitHub repository associated with this work (Borgqvist et al. 2023), and that a large emphasis has been put on writing reproducible code that is entirely open–source.

### Supplementary Information

Below is the link to the electronic supplementary material.

Supplementary file 1 (pdf 7923 KB)
